# Non-Financial Losses Incurred by a Traffic Accident Perpetrator

**DOI:** 10.1007/s13177-022-00329-x

**Published:** 2022-11-09

**Authors:** Hiroaki Miyoshi, Satoshi Taguchi, Tatsushi Yamamoto, Shoji Watanabe

**Affiliations:** 1 Faculty of Policy Studies, Doshisha University, Karasuma-higashi-iru, Imadegawa-dori, Kamigyo-ku, Kyoto, 602-8580 Japan; 2 Faculty of Commerce, Doshisha University, Karasuma-higashi-iru, Imadegawa-dori, Kamigyo-ku, Kyoto, 602-8580 Japan; 3 Institute for Technology, Enterprise and Competitiveness, Doshisha University, Karasuma-higashi-iru, Imadegawa-dori, Kamigyo-ku, Kyoto, 602-8580 Japan

**Keywords:** Traffic accident, Non-financial loss, Advanced driver-assistance system, Perpetrator

## Abstract

The drivers of vehicles equipped with active-safety systems are less likely to be the responsible parties in traffic accidents. However, this benefit cannot be monetarily evaluated because monetary values for non-financial losses incurred by perpetrators have thus far been entirely unexplored. This paper estimates the value using stated-preference survey. The main finding is that monetary value for non-financial losses incurred by a perpetrator of fatal accident (for cases in which the perpetrator was 100% responsible for the accident) should be set at the level approximately equal to that of non-financial losses to an accident victim.

## Introduction

Advanced driver-assistance systems (ADAS) and self-driving vehicles have been a growing focus of worldwide attention in recent years. These technologies are widely expected to furnish powerful tools for reducing or even eliminating traffic accidents, and many studies have been conducted with regard to the impacts of ADAS using retrospective assessment (e.g. [1, 2], and [3]) or prospected approach (e.g. [4–6], and [7]). (see [7]).

In contrast to airbags and other *passive* safety technologies that seek to minimize injury to passengers in the event of accidents, ADAS and autonomous-vehicle systems are *active* safety technologies: they seek not only to minimize harm from accidents, but to prevent traffic accidents from happening at all. Active safety technologies offer two benefits to users—and to society as a whole—that passive safety technologies cannot provide:

1. Because active-safety systems seek to avoid traffic accidents themselves, they reduce injuries not only for drivers of vehicles equipped with these systems, but also for the drivers of *other* vehicles and pedestrians (who experience fewer accidents thanks to the existence of the systems).
2. In view of point 1, drivers of vehicles equipped with active-safety systems are less likely to be the responsible parties in traffic accidents.

To date, economic evaluations of losses due to traffic accidents have been carried out in Japan and some other nations around the world. In addition to these financial losses, economic analyses of non-financial losses incurred by traffic-accident victims themselves—that is, pain and suffering experienced by individuals due to their own involvement in traffic accidents—have been conducted in some countries. The value of a statistical life (VSL) and the value of injury (VI) are necessary for evaluating this non-financial losses from traffic accident and the benefit from reducing traffic accident. This parameter has traditionally been measured using contingent valuation, chain method or standard gamble (e.g. [8–12], and [13]).

However, there remains one type of loss whose financial consequences have thus far been entirely unexplored: *non-financial losses to parties responsible for accidents*—that is, suffering associated with having caused an accident that exposed others to injury or death, as well as suffering associated with the concomitant social stigma and loss of trustworthiness within society. Among the reasons for this gap in the literature is that, because efforts to reduce traffic accidents previously relied on passive-safety technologies. However, in view of the growing adoption of active-safety technologies today, there is a pressing need to establish monetary values for non-financial losses incurred by accident causers to allow quantitative assessment of the benefits of point 2 above. Without this information, it is impossible to quantify the full benefit of installing road infrastructure, dynamic maps, and other provisions required for active-safety technologies, or the benefit of government policies to mandate installation of safety technologies in vehicles. In view of this situation, the objective of this study is to determine monetary values for non-financial losses incurred by causers of traffic accidents; as far as we are aware, this is the world’s first research study to attempt such a determination.

## Effectiveness of ADAS

The first step in our analysis is to evaluate the effectiveness of ADAS technologies in reducing traffic accidents. To this end, we first compute the total annual economic loss due to traffic accidents in Japan at 5-year intervals between 1992 and 2020, focusing on accidents between two four-wheel vehicles. Then we analyze how this loss varied over each 5-year interval and perform a factor decomposition to assess the relative contributions of various individual factors to the overall loss. Finally, we use the results of this analysis to quantify the role played by ADAS in reducing accidents.

More specifically, we restrict our analysis to accidents between 4-wheel vehicles, and we compute annual losses for each of 7 years (1992, 1997, 2002, 2007, 2012, 2017, 2020) in the form

1 $${S}_{n}={D}_{n}\cdot {A}_{n}\cdot {L}_{n}$$

where

*S*_*n*_: is the total economic loss due to traffic accidents in year *n* (1 ≤ *n* ≤ 7), computed by multiplying the number of accident victims in year *n* by the loss per individual victim established for accidents of the relevant severity level,
*D*_*n*_: is the total distance in kilometers traveled during year *n*,
*A*_*n*_: is the number of accident frequency per kilometer traveled for year *n*, and.
*L*_*n*_: is the economic loss per accident in year *n.*

Next, from (1) the variation in economic loss from year *n-1* to year *n* is related to the variations in other quantities according to

2 $$\frac{\Delta {S}_{n}}{{S}_{n}}=\frac{\Delta {D}_{n}}{{D}_{n}}+\frac{\Delta {A}_{n}}{{A}_{n}}+\frac{\Delta {L}_{n}}{{L}_{n}}+{R}_{n}$$

where e.g. ${\Delta S}_{n}\equiv {S}_{n}{-S}_{n-1}$ and the residual ${R}_{n}$ measures the portion of the variation in *S* that remains unexplained by the sum of the variations in its various factors. Equation (2) expresses variation rates of total economic loss as a sum of three contributions: variation rates of total kilometers traveled, variation rates of accident frequency per kilometer traveled, and variation rates of economic loss per accident.

**Fig. 1:**
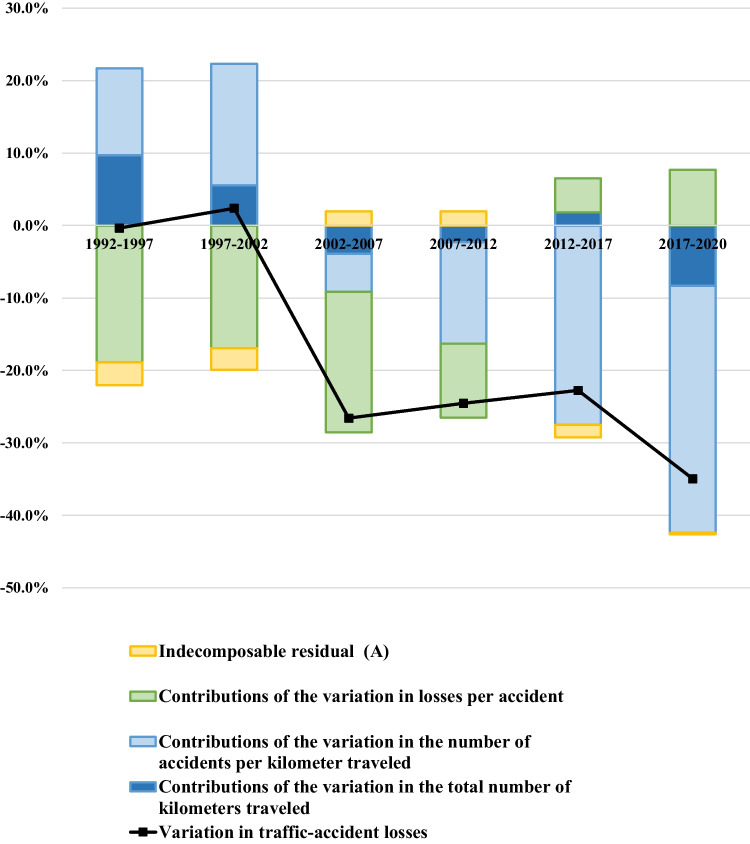
Factor-decomposition analysis of variations in traffic-accident losses. Note: 1. Analysis restricted to accidents between two four-wheel vehicles excluding special-purpose vehicles. 2. Calculated by author using J-TAD (macro) provided by ITARDA, losses per victim calculated from [14], and Monthly Statistical Report on Motor Vehicle Transport provided by MLIT

Results of this analysis are depicted graphically in Fig. 1. For example, comparing results for 1992 and 1997 shows that total economic loss increased by -0.4% over that 5-year interval. This -0.4% increase was the net result of three contributions: + 9.7% due to an increase in total kilometers traveled, + 12.0% due to an increase in accident frequency per kilometer, and -18.9% due to a decrease in loss per accident. (The residual, i.e. the portion of the overall variation that remained unexplained after accounting for variations in all factors, was -3.2%). In this case, increases in total kilometers traveled and in accident frequency per kilometer both had the effect of increasing overall loss, but these effects were partially offset by a decrease in loss per accident.

What are the relevant mechanisms responsible for driving three factors? First, variation in economic loss per accident may be due to the shifting composition (vehicle-type distribution) of automobile fleet; another possible driver is the increasing sophistication of passive-safety technology and its growing penetration into owned-vehicle fleet. On the other hand, variation in accident frequency per kilometer traveled may be due to increased driver awareness of safe-driving techniques, improvements in road travel environments, and advances in active-safety technologies—which can prevent accidents from happening at all—as these technologies also achieve penetration in privately-owned vehicles.

From Fig. 1 we see that the *variation in economic loss per accident* has the effect of reducing total economic loss in each of the 4 intervals between 1992 and 2012. In view of the fact that many standards for ensuring automobile collision safety were strengthened during this period, we can infer that advances in passive-safety technologies and their penetration into owned vehicles contributed significantly to enabling this development. On the other hand, this phenomenon then seems to vanish entirely, and indeed after 2012 this factor begins to work in the *opposite* direction, serving to increase losses due to traffic accidents. This may be in part attributed to the changes in the vehicle-type distribution of automobile fleet during this time period.

Meanwhile, the *variation in accident frequency per kilometer traveled* has the effect of *increasing* total traffic-accident losses before 2002, but crosses over to *reducing* total losses after 2002, with the effect becoming particularly significant in recent years. This behavior is considered to be partially due to the penetration of active-safety technologies into owned vehicles.^1^

We also note that, between 2017 and 2020, the factor *change in total kilometers traveled* serves to decrease total traffic-accident losses. We attribute this to the massive decrease in transit volumes for 2020 due to the COVID-19 pandemic.

## Study Design

The magnitude of non-financial losses incurred by traffic accident *perpetrator* varies significantly depending on a range of factors, including the extent of the perpetrator’s fault or negligence, the number of victims, and the severity of bodily injuries of victims resulting from the accident. Non-financial losses also presumably differ for vehicle-vehicle and vehicle–pedestrian accidents. For specificity, in this study we restrict attention to accidents satisfying the following conditions: the accident involves two vehicles, each with one human driver and no other passengers; the accident is entirely due to fault or negligence on the part of one driver (the *perpetrator*), i.e. the perpetrator bears 100% responsibility for causing the accident, while the other driver (the *victim*) bears 0% responsibility; finally, the perpetrator suffers only slight injuries, with no residual disability from the accident, while the victim dies in the accident. After refining the survey methods used for our preliminary surveys based on methods of experimental economics, we conducted online stated preference (SP) surveys in which respondents were asked to envision being the perpetrator of such an accident and queried to assess their willingness to pay for safety devices that reduce the probability of such accidents; we refer to these surveys as *perpetrator surveys.*

The results of perpetrator surveys contain information regarding non-financial loss to accident perpetrator; to understand this information in full context, it must be complemented by corresponding information regarding non-financial loss to accident *victim.* Thus we complement our perpetrator surveys by conducting *victim surveys*, which are nearly identical to perpetrator surveys except with respondents now taking the perspective of accident victims.^2^

### SP Survey

In this section we discuss our procedures for conducting the perpetrator and victim surveys described above.^3^ SP surveys were conducted over a two-day period, April 27–28, 2021. Survey respondents were required to hold ordinary driving licenses valid throughout Japan. Respondents were assigned randomly to participate in perpetrator or victim surveys. After eliminating inappropriate respondents including those who seemed lacking in understanding, our analytical sample set contained responses from 2,085 respondents. Statistics on the genders, ages, household incomes, and group numbers of respondents are tabulated in Table 1.

**Table 1:** Demographic characteristics of survey respondents

Variable	Value	Perpetrator surveys		Victim surveys	
		Sample size	Ratio	Sample size	Ratio
Gendar					
	Female	518	49.8%	511	48.9%
	Male	522	50.2%	534	51.1%
Age (years)					
	18–39	350	33.7%	355	34.0%
	40–59	352	33.8%	348	33.3%
	60 +	338	32.5%	342	32.7%
Traffic accident reduction rate for the first question					
	50%	527	50.7%	523	50.0%
	90%	513	49.3%	522	50.0%
Net Income (Household)					
	Less than 5 mil. JPY	500	48.1%	498	47.7%
	No less than 5 mil. JPY and less than 10 mil. JPY	420	40.4%	402	38.5%
	No less than 10 mil. JPY	120	11.5%	145	13.9%

All surveys followed the same basic outline: First, respondents were provided with several pieces of information describing a hypothetical traffic accident and a new driving-safety system that reduces the likelihood of such accidents (Section 3.1.1). Then respondents were asked a sequence of 4 yes-or-no questions to determine their willingness to pay for the new safety system (Section 3.1.2).

#### Information Provided to Respondents

Each survey began by providing the respondent with various pieces of information, as follows:

##### Description of Accident Scenario

**Fig. 2:**
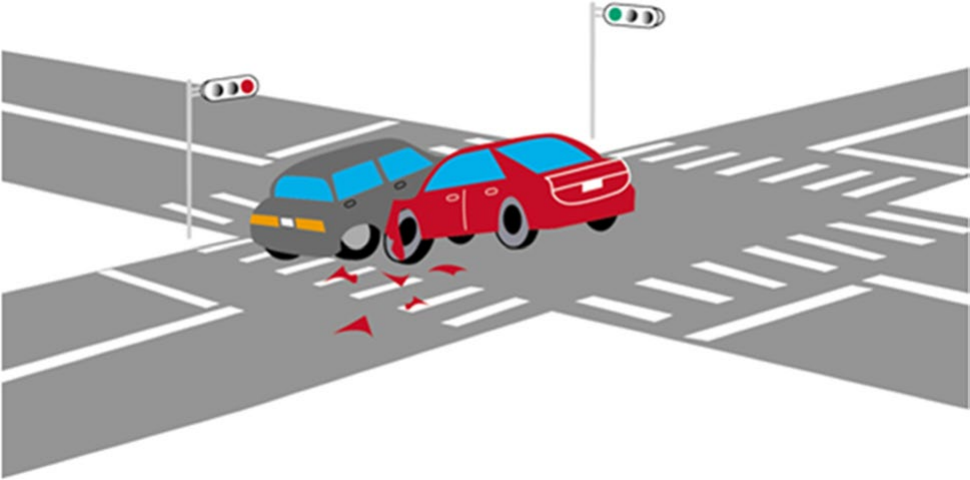
Illustration of hypothetical vehicle-vehicle accident shown to survey respondents. Source: University of Tokyo and Doshisha Univ. [15]

All survey respondents were shown the image of Fig. 2 and asked to consider a fatal vehicle-vehicle collision accident like that shown in the image. For *perpetrator* survey, respondents were asked to picture themselves as the driver of the red car; for *victim* survey, respondents were asked to picture themselves as the driver of the grey car. In both cases, respondents were told that the driver of the grey car immediately loses consciousness and dies as a result of the accident. Although perpetrator surveys to assess non-financial losses to perpetrators may be influenced by factors such as whether the victim is a child or adult, our surveys did not incorporate special provisions to address these points.

##### Description of Consequences for Accident Perpetrators and Victims

Perpetrator survey respondents were informed that they would be personally subject to a host of consequences, including criminal liability, civil liability, administrative consequences, ethical/societal ramifications and other consequences. Along with this, respondents were informed that insurance coverage would ensure that neither they nor their families would incur financial losses due to the accident.

Victim survey respondents were informed that, because of insurance coverage and other factors, neither they nor their families would incur any financial losses due to the accident.

##### Recent Statistics on Traffic-Accident Frequency in Japan

Respondents were informed that, as of 2018, one out of every 200,000 licensed drivers in Japan dies each year in an accident involving two 4-wheel vehicles. To offer a sense of the magnitude of this risk of causing an accident (for perpetrator survey) or suffering an accident (for victim survey), respondents were told that a probability of 1-in-200,000 corresponds to approximately one-half the probability of dying in a fire.

##### Description of New Driving-Safety System

Finally, survey respondents were informed of a newly developed driving-safety technology system available for drivers to purchase. The only detail provided to respondents about this system was its efficacy rate, defined as the fraction of traffic accidents avoided with the system installed. For this rate we considered values of 50% and 90%; as discussed below, each respondent was asked to consider both possibilities, in either ascending or descending order, as part of our strategy for using yes-or-no questions to elicit willingness-to-pay values. This is described in the following section.

#### Questions Asked of Respondents

Having provided respondents with information on hypothetical accidents and a safety system capable of avoiding them, we sought to determine the prices respondents would be willing to pay for the system at efficacy rates of 50% and 90%. (Here “price” refers to the cost of using the system for one year.) To avoid bias due to the order in which these rates were considered, respondents were randomly divided into two groups: *Group 1* respondents were first asked to consider 50%-effective systems, then to consider 90%-effective systems; *Group 2* respondents were first asked to consider 90%-effective systems, then to consider 50%-effective systems.

Respondents were asked to state their WTP using double-bounded dichotomous choice: respondents were not asked their WTP directly, but were asked a total of 4 yes-or-no questions (two for each efficacy rate) as follows (considered here for respondents in Group 1):

- Question 1: Respondents were presented with an initial price chosen at random from a set of 5 prices ({1, 5, 10, 30, 50} thousand yen) and asked if they would be willing to purchase a 50%-effective safety system at this price. The 7 price values shown in Table 2 are the values used in surveys by the Cabinet Office (2017); we conducted preliminary surveys to confirm that this range of values reflects the distribution of willingness-to-purchase prices for victims.
- Question 2: As shown in Table 2, respondents answering *yes* to question 1 were then asked the same question at the next higher price in the set; respondents answering *no* were asked at the next *lower* price in the set. For example, if the randomly-selected initial price for Question 1 was 10,000 yen, the price for Question 2 would be 30,000 yen for respondents answering *yes* to question 1 and 5,000 yen for respondents answering *no*.
- Questions 3 and 4: Like questions 1 and 2, but now for a 90%-effective safety system.

**Table 2:** Prices presented to respondents in sequence of two yes-or-no questions asked to determine willingness to pay for safety-system devices

Initial price	Answer for initial price	Second price
1,000	Yes	5,000
	No	100
5,000	Yes	10,000
	No	1,000
10,000	Yes	30,000
	No	5,000
30,000	Yes	50,000
	No	10,000
50,000	Yes	100,000
	No	30,000

The same table was used for Questions 1 and 2 and for Questions 3 and 4

The procedure for Group 2 respondents was similar, but now with questions 1 and 2 regarding the 90%-effective system and questions 3 and 4 regarding the 50%-effective system.

In our parameter-estimation described below, for each individual survey respondent we constructed a dataset containing 6 combinations of price and yes-or-no answer. Four out of 6 combinations were each respondent’s answers (two for each efficacy rate). Remaining two out of 6 combinations were generated combination for each efficacy rate under the assumption that survey respondents behave rationally. For respondents answering *yes* to question 1, combination of the next lower price and answer-yes were generated. For respondents answering *no* to question 1, combination of the next higher price and answer-no were generated. This method is for avoiding sampling bias.

### Analytical Method

Here explanatory variables in the binary logistic regression model and their expected sign in regression coefficients are explained in Section 3.2.1. The methodology for scope test is also described in Section 3.2.2.

#### Explanatory Variables

For both perpetrator and victim surveys, we considered binomial logit models with *willingness to pay for safety devices* as a binary response variable taking values of 1 (willing to pay) or 0 (not willing to pay). We then performed parameter estimation to estimate values for a host of explanatory variables in an indirect utility function. These explanatory variables are as in Table 3. The expected coefficient sign for each variable is briefly described below.

**Table 3:** Explanatory variables in binomial logit model

Variable	Description
Price	Price for 1 year’s use of safety device (100 | 1,000 | 5,000 | 10,000 | 30,000 | 50,000 | 100,000 JPY)
Reduction_rate	Efficacy rate of safety device in reducing accident likelihood (50% | 90%)
Accident_experience_dummy	Whether or not respondent has personally experienced a traffic accident (1: yes | 0: no)
Aged_70_ dummy	Age (1: 70 or above | 0: below 70)
Confidence_driving_dummy	Confidence in driving (1 for respondents who answered “extremely confident” or “confident” | 0 for respondents who answered “not very confident,” “not confident at all”, or “neither”)
Distance_10thousandKm_dummy	Total driving distance in 2020 (1: 10,000 km or more | 0: less than 10,000 km)
Household_income	Annual household income in 2020 (0.5: below 1 million yen |1.5: not less than 1 million yen and less than 2 million yen | 2.5 | 3.5 | 4.5 | 5.5 | 6.5 | 7.5 | 8.5 | 9.5 | 10.5 | 11.5 | 12.5 | 13.5 | 14.5 | 15.5 | 16.5 | 17.5 | 18.5 | 19.5 | 20.5: above 20 million yen)
Household_income_dummy	1: Annual household income in 2020 is 20 million yen or above | 0: under 20 million yen
ADAS_experience_dummy	Whether or not respondents have previously used one of the following four ADAS technologies: (collision-mitigation brakes (automatic brakes), devices to limit acceleration when accelerator pedal is pressed by accident, warning systems to alert drivers to lane deviations, or adaptive cruise control (1: yes | 0: no)
Risk_coef.	Relative degree of risk aversion coefficient
Risk_coef._dummy	1: respondent who choose Lottery B at the question of (*p*, 1—*p*) = (0.9, 1) for the first time | 0: others

##### Variables Expected to have Positive Coefficients in both Perpetrator and Victim Surveys

*Reduction_rate*: The greater the efficacy of the safety device in preventing accidents, the greater the benefit it provides to drivers, and hence the greater the price drivers will presumably be willing to pay.

*Accident_experience_dummy*: Respondents who have personally experienced traffic accidents will presumably have a greater intuitive appreciation for the costs of perpetrating or suffering traffic accidents.

*Household_income* and *Household*_*income_dummy*: In both surveys, respondents reported household income by responding to a multiple-choice question, with only one choice for income of 20 million yen or above. The variable of *Household_income_dummy* is added to eliminate the influence of this effect from household income. Respondents with higher household incomes presumably derive more benefit from avoiding traffic accidents.

*Risk_coef.* and *Risk_coef._dummy*: To assess respondents’ relative degree of risk aversion, we used a version of the lottery-choice scheme discussed by [17]. For various probabilities *p*, respondents were asked to select their preference of two lottery schemes:

Lottery A: Win 20,000 yen with probability *p*;

otherwise (with probability 1 - *p* ) win 16,000 yen.

Lottery B: Win 38,500 yen with probability *p*;

otherwise (with probability 1 - *p* ) win 1,000 yen.

Respondents were first asked their preference for *p* = 0.1. If the respondent chose Lottery A, the question was asked again for *p* = 0.2. This process continued, with *p* increasing through the sequence {0.1, 0.2, …, 0.9, 1}, until the respondent selected Lottery B. Then, using the function u (*x*) = *x* ^(1 − *γ*)^ / (1—*γ*) where *x* is winning amount, we compute the minimum value of γ with three decimal places for which the expected utility for Lotteries B is greater than that of A, then take the respondent’s relative degree of risk aversion to be equal to the γ value for the first question on which the respondent chose Lottery B. It is impossible to calculate the relative degree of risk aversion coefficient when a respondents choose Lottery B at the question of (*p*, 1—*p*) = (1,0) for the first time. In this case, *Risk_coef.* is set equal to the case for a respondent who choose Lottery B at the question of (*p*, 1—*p*) = (0.9, 1) for the first time and *Risk_coef._dummy* is set to 1.

Respondents with higher degrees of risk aversion place a greater premium on avoiding risks and are thus presumably willing to pay more for safety devices that reduce the risk of traffic accidents.

*Distance_10thousandKm_dummy*: The greater the distance traveled, the greater the benefit derived by avoiding accidents, and thus the greater the price respondents are willing to pay for safety devices.

##### Variables Expected to have Positive Coefficients in Perpetrator Survey

*Aged_70_dummy*: In recent years, traffic accidents caused by older adult drivers have been a growing problem in Japan. For this reason, older adult survey respondents presumably have greater fears of causing accidents than respondents in other age brackets and thus should presumably be willing to pay more for safety devices.

*ADAS_experience_dummy*: Respondents who have previously used one or more of the various safety systems that are currently commercially available will presumably have a greater intuitive appreciation for the usefulness of such systems.

##### Variables Expected to have Negative Coefficients in both Perpetrator and Victim Surveys

*Price*: The higher the price of the safety system, the fewer respondents who will presumably be willing to purchase it.

##### Variables Expected to have Negative Coefficients in Perpetrator Survey

*Confidence_driving_dummy*: Respondents with confidence in their own driving skill will presumably derive less benefit from safety devices intended to reduce their likelihood of causing accidents.

Parameter estimation for our logit model was performed in R using the *glm* generalized linear model with the binomial (logit) response function.

#### Scope Test

The technique used in this study to assess willingness to pay for safety devices is an example of a *contingent-valuation method* (CVM). Scope test is a technique for assessing the reliability of CVM results ([18–20], and [21]).

Our SP surveys asked respondents to consider events that are already extremely rare—traffic accidents occurring with a probability of just 1 in 200,000—and attempt to gauge the willingness of respondents to pay for devices that may further reduce these tiny probabilities by 50% or by 90%. Clearly, we require a validation step to test whether or not respondents are correctly interpreting these small variations in minuscule probabilities and reflecting this understanding in their survey responses. We implement such a validation step by conducting scope tests referring to the method in [22].

Scope tests include both *internal* scope tests, which consider differences in a single respondent’s willingness to pay to avoid two different types of risks, and *external* scope tests, which consider differences between the willingness of *different* respondents to respond to different types of risks. For this study we designed four scope tests—two internal and two external—and applied all four tests to the results of both perpetrator and victim surveys.

The common basis for all of our scope tests is a binomial logit model with two explanatory variables: *Price* and *Reduction_rate*. Each individual scope test proceeds by defining a particular subset of interest within the full set of survey data, and then, performing parameter estimation for the binomial logit model *using only the specified subset of survey data.* The test passes if this parameter estimation produces a positive coefficient for the Reduction_rate.

The data subsets used for our four scope tests are:

- *Data subset I*: Survey results for Group-1 respondents only,
- *Data subset II*: Survey results for Group-2 respondents only,
- *Data subset III*: Survey results for all respondents regarding only the *first* of the two efficacy rates respondents were asked to consider—that is, responses from Group-1 respondents regarding 50%-effective systems and responses from Group-2 respondents regarding 90%-effective systems,
- *Data subset IV*: Survey results for all respondents regarding only the *second* of the two efficacy rates respondents were asked to consider—that is, responses from Group-1 respondents regarding 90%-effective systems and responses from Group-2 respondents regarding 50%-effective systems.

Of these, data subsets I and II correspond to internal scope tests, while data subsets III and IV correspond to external scope tests.

## Results

The results of scope tests are as follows: the coefficients for *Price* are negative and statistically significant at the 5% level and coefficients for *Reduction_rate* are positive and significant at the same level, for all data subsets in both perpetrator and victim surveys. Our scope tests produced good results overall. The results of external scope test are as shown in Table 4.

**Table 4:** Results of logit model estimation

		Perpetrator surveys				Victim surveys			
		Model 1 (External scope test)		Model 2		Model 3 (External scope test)		Model 4	
		Est. value (z-value)		Est. value (z-value)		Est. value (z-value)		Est. value (z-value)	
Intercept		1.679	*	1.548	*	1.060	*	0.861	*
		(9.88)		(7.76)		(6.69)		(4.79)	
βPrice		-0.0000546	*	-0.0000550	*	-0.0000528	*	-0.0000541	*
		(-22.6)		(-22.5)		(-21.4)		(-21.5)	
βReduction_rate		0.00461	*	0.00395		0.00973	*	0.00963	*
		(2.03)		(1.71)		(4.44)		(4.34)	
βAccident_experience_dummy				-0.152				-0.326	*
				(-1.61)				(-3.56)	
βAged_70_dummy				0.446	*			0.303	
				(2.56)				(1.69)	
βConfidence_driving_dummy				-0.157				-0.103	
				(-1.44)				(-0.975)	
βDistance_10thousandKm_dummy				-0.344	*			-0.117	
				(-2.13)				(-0.721)	
βHousehold_income				0.0191				0.0418	*
				(1.46)				(3.381)	
βHousehold_income_dummy				1.48	*			-1.27	*
				(2.34)				(-3.43)	
βADAS_experience_dummy				0.087				0.454	*
				(0.846)				(4.52)	
βRisk_coef.				0.187	*			-0.02665	
				(2.94)				(-0.4359)	
βRisk_coef._dummy				0.164				0.151	
				(1.25)				(1.241)	
Model Fit									
Log likelihood (null model)		-1911.790		-1911.790		-1970.328		-1970.328	
Log Likelihood		-1503.747		-1481.121		-1610.037		-1584.128	
McFadden R2		0.213		0.225		0.183		0.196	
Estrella R2		0.745		0.731		0.776		0.760	
Likelihood ratio test	χ^2^	816.087		861.339		720.581		772.400	
	p-value	0.000		0.000		0.000		0.000	

* denotes not significant at a 95%—level

Next, we perform parameter estimation for the variables of Table 3 in the binary logit model. The data set we use for this purpose is data subset III. The reasons for this is that previous studies asking multiple WTP questions in a single survey have reported *ordering effects*: reported WTP values tend to be lower for questions asked later ([23]). Data subset III contains only responses to the first WTP question asked of respondents and is thus immune from such ordering effects.

Results are presented in Table 4. For both perpetrator and victim surveys, the coefficients for *Reduction_rate* are positive and the coefficients for Price are negative, but *Reduction_rate* is not significant for perpetrator survey.

Next, to our surprise, the coefficient for *Accident_experience_dummy* was negative and significant for victim survey. One possible explanation for this finding is as follows: Most traffic accidents result in slight injuries, and respondents whose accident experience produced only slight injuries and was, moreover, adequately reimbursed by insurance coverage may—counter to expectation—feel less need than other respondents to install safety devices.

As expected, the coefficient for Aged_70_dummy is positive and significant for perpetrator survey. For victim survey, one might have expected the coefficient for this variable to be negative because the older adult derive less benefit than other age brackets from avoiding traffic accidents; however, in fact, the coefficient of *Aged_70_dummy* is positive but not significant.

As expected, the coefficients for *Confidence_driving_dummy* were negative but not significant for both surveys.

In contrast to expectations, the coefficient for *Distance_10thousandKm_dummy* was negative; this variable was significant for perpetrator survey, but not significant for victim survey. The influence of personal experience with traffic accidents is absorbed by the coefficient of *Accident_experience_dummy*, so the coefficient for this variable should reflect willingness to pay for safety devices for long journeys *without* accident experience—but long drives without accidents may have the effect of weakening the sense of urgency for installing safety devices. This effect would presumably be larger for perpetrators than for victims.

As expected, the coefficients for *Household_income* and *ADAS_experience_dummy* are positive for both surveys, but significant only for victim survey.

Finally, as expected, the coefficients of *Risk_coef.* were positive for both surveys, but significant only for perpetrator.

We next compute the median and average WTPs for safe-driving devices. Here we use estimation results obtained with scope-test Dataset III (Models 1 and 3) to compute median and mean values for the annual price of safety-device use.

Results are presented in Table 5. Because average WTPs tend to get elevated to abnormally high values in the presence of one or more survey respondents with very high WTPs, the median WTP is often the more appropriate choice for applications to government policy and will be our focus here. For perpetrator survey, we find median WTPs of 35,005 yen for 50%-effective devices and 38,388 yen for 90%-effective devices. For victim survey, we find slightly lower WTPs of 29,266 yen for 50%-effective devices and 36,631 yen for 90%-effective devices.

**Table 5:** Mean and median of willingness-to-pay (WTP) values (Yen)

	Perpetrator survey (a)	Victim survey (b)	a/b
Estimated median for 50% reduction	35,005	29,266	1.20
Estimated median for 90% reduction	38,388	36,631	1.05
Estimated average for 50% reduction	37,536	32,919	1.14
Estimated average for 90% reduction	40,516	39,181	1.03

The median WTP in perpetrator survey is thus 1.20 times higher than that in victim survey (for 50%-effective devices) or 1.05 times higher for 90%-effective devices. These are higher ratios than we expected to find when we began this work, and we suspect this finding may be related to the phenomenon of traffic accidents caused by older adult drivers, which—as we noted above—has recently become a significant problem in Japan.

Non-financial losses for victims and for perpetrators are estimated by dividing device WTP values by accident reduction rates and accident occurrence probabilities. We note that the value of 537 million yen computed by [14] for non-financial losses to victims will be used in cost–benefit analyses at both the national and regional level. Consequently, our strategy of multiplying this value by the perpetrator-to-victim WTP ratios in Table 5 to compute non-financial losses for perpetrators may be considered appropriate for use in establishing actual policies. Using median values for this computation yields non-financial losses for perpetrators of 642 (for 50% reduction rate) and 563 (for 90% reduction rate) million yen.

## Conclusions and Future Work

In this study, we first quantified the effectiveness of advanced driving-assistance systems in reducing the harm caused by traffic accidents—and, in so doing, underscored the importance of establishing monetary values for non-financial losses incurred by the perpetrators of such accidents. Then we outlined our procedure for conducting SP surveys and analyzing the results to estimate values for these losses. Based on the results of this analysis, we concluded that monetary values for non-financial losses to perpetrators of fatal accidents (for cases in which the perpetrator was 100% responsible for the accident) should be set at levels approximately equal to those of non-financial losses to accident *victims.* As noted above, non-financial losses to accident perpetrators depend on many factors, including: the extent of the perpetrator’s fault or negligence; the number of victims, and the severity of their bodily injuries; and whether the accident involved vehicle-vehicle or vehicle–pedestrian altercations. In view of these complications, in future work we plan to extend this study in at least seven directions.

First, we will expand consideration to a broader variety of accidents. This study considered only vehicle-vehicle accidents, but studies of vehicle–pedestrian accidents are also needed; we expect that non-financial losses to perpetrators of such accidents may be even greater than in the vehicle-vehicle case.

Second, whereas this study considered only accidents resulting in fatalities, we will expand consideration to non-fatal accidents. New SP surveys involving non-fatal accident scenarios will be required to conduct.

Third, we will assess how non-financial losses to perpetrators vary depending on particular attributes of the perpetrators or the victims of the accidents they cause. For example, non-financial losses to perpetrators may vary significantly depending on whether the victim is a child or adult and/or family composition of the victim.

Fourth, we will consider the effect of restricting surveys or analyses to include only respondents with direct personal experience of involvement in traffic accidents. We expect that WTP values for such traffic-accident survivors, and the corresponding non-financial loss values, may yield more accurate financial estimates of the psychological burden experienced by accident perpetrators.

Fifth, we will employ more sophisticated survey techniques. The SP surveys conducted for this study used illustrations to describe accidents, but the use of driving simulators to allow survey participants to experience accidents virtually may yield more accurate estimates of WTP values and non-financial losses.

Sixth, we will consider non-financial losses to accident perpetrators as a contribution to overall traffic-accident losses in Japan. To date, Japan’s Cabinet Office has conducted several analyses in which traffic-accident losses were evaluated financially and presented in reports including [14]. Adding those contributions and characterizing the year-by-year evolution of traffic-accident losses is a key priority for future work.

Seventh, we will analyze the benefits of active-safety devices including the reduced non-financial losses to perpetrators. The results will constitute a valuable basic resource for evaluating policy initiatives related to passive-safety systems, such as regulations mandating the installation of safety devices.

## Footnotes

1 The data used for this analysis consist of statistics on traffic accidents reported to the police as human-casualty accidents. However, [16] notes that, since the mid-2000s, an increasing number of accidents have been reported to the police as involving only property damage despite in fact involving human casualties. This underreporting of human-casualty accidents may be one cause of the decrease in accidents per travel distance observed in Fig. 1.

2 Non-monetary losses to accident victims could be extracted from the findings of [14]; however, the accident scenarios and accident occurrence probabilities considered in [14] differ from those used in our victim survey, and thus cannot be directly compared to the results of our victim survey.

3 Contents of SP surveys in this study are described in detail in [15]
